# Mental distress and its association with sociodemographic and economic characteristics: community-based household survey in Aceh, Indonesia

**DOI:** 10.1192/bjo.2020.108

**Published:** 2020-11-04

**Authors:** Anna Reuter, Sebastian Vollmer, A. Aiyub, Suryane Sulistiana Susanti, M. Marthoenis

**Affiliations:** Department of Economics & Centre for Modern Indian Studies, University of Göttingen, Germany; Department of Economics & Centre for Modern Indian Studies, University of Göttingen, Germany; Department of Psychiatry and Mental Health Nursing, Universitas Syiah Kuala, Banda Aceh, Indonesia; Department of Community Nursing, Universitas Syiah Kuala, Banda Aceh, Indonesia; Department of Psychiatry and Mental Health Nursing, Universitas Syiah Kuala, Banda Aceh, Indonesia

**Keywords:** Mental distress, common mental disorder, socioeconomic, family functioning

## Abstract

**Background:**

The role of sociodemographic and economic characteristics in mental distress has been rarely investigated in Indonesia.

**Aims:**

To investigate the prevalence of common mental disorders (CMD) and identify any associations between mental distress and sociodemographic and economic characteristics among communities living in urban and rural (peri-urban) areas.

**Method:**

A community-based household survey was conducted in the province of Aceh, Indonesia, in 2018. The 20-item Self Reporting Questionnaire (SRQ-20) screening tool was used to measure symptoms of CMD. Information on sociodemographic characteristics, family functioning, labour market outcomes and healthcare costs was collected. Multivariate regressions were conducted to analyse the relationships between the measures of mental distress and sociodemographic and economic characteristics.

**Results:**

We found that 14% of the respondents had CMD symptoms. SRQ-20 scores were higher for female, older and lower-educated individuals. CMD prevalence was higher among non-married participants and clustered within families. Participants with CMD perceive their families as performing significantly better in the dimensions of affective involvement and behaviour control compared with their counterparts. Their work was more often affected by negative feelings; they were also twice as likely to report a recent physical or mental health complaint and faced twice the treatment costs compared with their non-affected counterparts.

**Conclusions:**

The prevalence of mental disorders is especially high in disadvantaged population groups. Moreover, mental distress is associated with a lower perceived productivity and a higher physical health burden.

Depressive and anxiety disorders belong to the ten largest global contributors to years lived with disability (YLD).^1^ In 2016, they each had a worldwide prevalence of 250 million cases.^1^ Despite this large disease burden, substantial treatment gaps exist, especially in low- and middle-income countries (LMICs); population-based studies have revealed that less than 10% of all people with depression and less than 3% of all people with anxiety disorders receive adequate care in LMICs.^2,3^

This treatment gap is particularly worrisome as depressive and anxiety disorders, also referred to as common mental disorders (CMD), can have detrimental impacts on further facets of well-being. CMD are associated with poor physical health and predict the onset of chronic physical conditions.^4,5^ Studies have found that people with CMD have worse role functioning and their families show poorer family functioning.^4,6–8^ Finally, the financial burden of CMD can be substantial: a recent review found that adults with depression face on average 2.6 times the direct healthcare costs and 2.3 times the indirect costs of their non-depressed counterparts.^9^ However, all included studies were conducted in high-income countries; comparable data for LMICs are widely missing or focus on poverty proxies rather than costs.^10^ One exception are the World Mental Health Surveys, which show a negative correlation for serious mental illnesses with unemployment and earnings.^4,11^

This data gap also exists in Indonesia. Although some studies have investigated which sociodemographic groups are most affected by CMD, the evidence on costs associated with mental disorders is thin.^12–15^ Two studies found a significant correlation between unemployment and poor mental health;^13,15^ another two found a negative association between expenditure levels and mental health,^13,14^ while yet another found no significant association for expenditure.^12^ A detailed analysis of the possible cost structure of mental illnesses is missing in all of these studies.

This study focuses on the province of Aceh, Indonesia, where the past 15 years have seen efforts to improve the situation of people with mental disorders. The mental healthcare system was shifted towards community mental health services^16,17^ and policies were initiated to end traditional practices of physical restraint and confinement (*pasung*).^18^ Although the prevalence of CMD in Aceh has historically been high,^19,20^ in 2018 it lowered to 9% (close to the national average).^21^ Still, previous research suggests that stigmatisation and traditional perceptions of mental illness might lead to a low rate of case detection and thus to undertreatment.^22^

This study aims to draw a comprehensive picture of CMD in Aceh. First, the overall prevalence and the affected populations were identified. Second, the association of CMD with health and economic factors was analysed to evaluate the potential triple burden of CMD, poor health and worse financial outcomes.

## Method

### Data collection

**Fig. 1:**
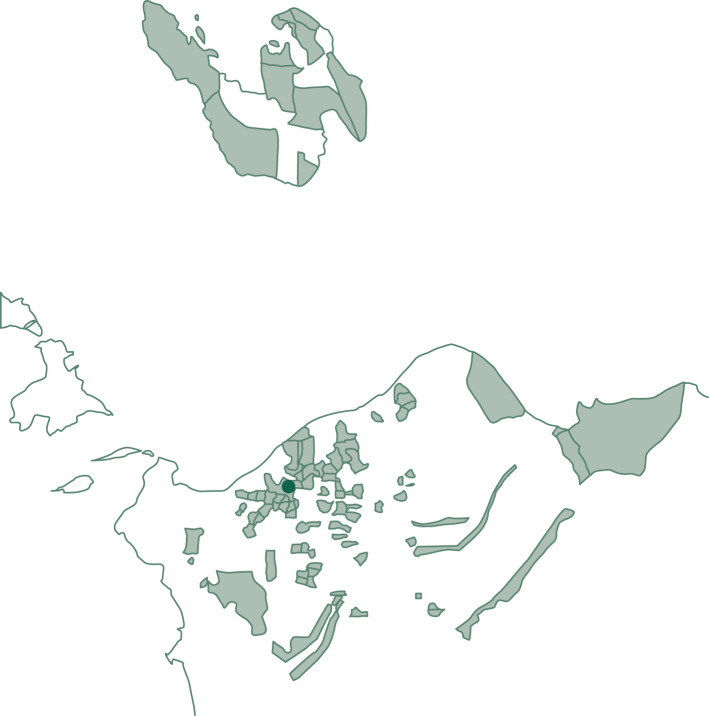
Villages in the study sample. Administrative areas of the villages included in the study sample (universal transverse Mercator (UTM) projection zone 46) are depicted in light green. The location of the Baiturrahman Grand Mosque in the city centre of Banda Aceh is marked in dark green.

From February to April 2018, we conducted a cross-sectional household survey in the district of Aceh Besar (the following districts were excluded for logistical reasons: Pulo Aceh, Lhoong, Lembah Seulawah, Leupung and Kota Jantho) and the cities of Sabang and Banda Aceh in Aceh province, Indonesia. Within each district or city, subdistricts were randomly sampled using population weights based on population data from the regency's statistical office.^23,24^ Within the subdistricts, villages were randomly sampled. The approached villages are identified in Fig. 1. Within the villages, households were sampled using a random walk scheme; if a sampled household was absent, it was visited again at another date and time. Within each household, all members aged 17 years and older were asked to participate in the individual interview (the key informant of the household identified household members). Additionally, one member (preferably the household head/spouse) was interviewed for the general household questionnaire. Written informed consent was obtained from each participant; refusal was possible for the complete survey as well as for each single item. The interviews were conducted by nursing students from Syiah Kuala University and the Nursing Academy Sabang, who undertook a 2-day training prior to data collection. Ethical approval was obtained from Syiah Kuala University and the University of Göttingen.

### Measurement of CMD

To measure CMD, we used the 20-item Self Reporting Questionnaire (SRQ-20).^25,26^ The SRQ-20 was introduced by the World Health Organization (WHO) to screen for non-psychotic disorders in LMICs and has been widely implemented,^25–28^ including in the study region.^29^ The original instrument consists of 20 questions regarding the prevalence of somatic, cognitive and emotional symptoms over the past 30 days, measured 0 or 1 (No/Yes), thus adding up to a 0–20 scale. To adjust the SRQ-20 to local norms, we dropped the question on suicidal thoughts. An earlier study in Indonesia validated the full SRQ-20 using a 5/6 cut-off (SRQ-20 ≥ 6) to indicate the prevalence of CMD (positive predictive value 60%, negative predictive value 92%);^30^ this cut-off is also applied by the health surveys from the Indonesian Ministry of Health. We used this cut-off despite removing one item, thus obtaining a rather conservative estimate. The Cronbach alpha of the SRQ-20 in the present study was 0.813, which suggests a good internal consistency. The single items are depicted in supplementary Table 1, available online at https://doi.org/10.1192/bjo.2020.108.

### Further household and individual characteristics

The household and individual characteristics used for the analyses are shown in supplementary Table 2. Gender, age and years of education served as controls in the main analyses, and we used information on marital status, occupation, district and reception of any state support to assess possible differences in the prevalence of mental distress across these groups. Reception of any state support is a variable collected at the household level and indicates whether any member of the household was a beneficiary of one of six public social support programmes (Raskin, KIS, JKA, BSM, PKH and KKS).

Family functioning was measured using a shortened version of the McMaster Family Assessment Device (FAD).^31^ The items are grouped into six categories adapted from the McMaster Model of Family Functioning: problem-solving, communication, roles, affective responsiveness, affective involvement and behaviour control, plus an extra category for general functioning. Each item asks for the level of agreement with a statement regarding one of these dimensions, ranging from ‘strongly disagree’ (1) to ‘strongly agree’ (4).

We assessed associated costs by analysing potential indirect costs (labour force participation, absenteeism, presenteeism) and potential direct costs (costs of treatment seeking), similar to König et al (2019).^9^ Labour-related characteristics include data on employment status, weekly working hours and days as well as monthly payment. Moreover, we assessed the subjective impact of mental distress on work productivity: we specifically asked whether participants’ work in the past 30 days had been affected by negative feelings, how many days of work had been affected, whether physical problems had been the reason for the negative feelings and whether the individual had sought treatment owing to these feelings.

For the assessment of healthcare costs, we used self-reported information on the occurrence of any health complaints (physical or mental) over the past 30 days; whether treatment was sought and at which type of facility (multiple answers possible); how much time was spent on travelling, waiting and the treatment; and how much money was spent on travelling, treatment and medication.

### Statistical analysis

We regressed the scores of the SRQ-20 on age, gender and education to analyse the association of mental distress with these base characteristics using an *F*-test for joint significance. In the subsequent steps, we use these base characteristics as controls.

To identify which socioeconomic groups were affected, we employed a linear probability model regressing the indicator for CMD (SRQ-20 ≥ 6) on the respective socioeconomic characteristics, controlling for age, gender and education and then predicting the prevalence of CMD over the groups of interest. To assess the association of family functioning with CMD, we used an ordered logistic model regressing each FAD item on the CMD indicator, controlling for age, gender and education. Finally, to analyse the costs associated with CMD, we regressed the outcome of interest on the CMD indicator, controlling for age, gender and education, and then predicted the outcome by CMD status. All models clustered standard errors at the household level. All statistical analyses were conducted with Stata/SE 15.1 on MacOS.

## Results

### Participation rates

Overall, 821 households were approached; 640 (78%) agreed to participate, 132 were absent or busy, and the remainder refused or did not participate for other reasons. On average, 2.47 interviews were conducted per household. In total, the participating households consisted of 2107 adults, of whom 1490 (71%) were present and agreed to participate. In eight cases, the household questionnaire (but no individual interview) was completed; these cases were excluded in the following analysis.

### Descriptive statistics

Table 1 shows sociodemographic characteristics as well as the SRQ-20 results for the respondents. Our sample consisted of 62% women; 40% of the participants reported current employment. Interestingly, 92% reported having health insurance, indicating that universal healthcare coverage, as planned by the Indonesian government, is in feasible reach in the study region. Nearly half of the respondents (43%) reported that they had experienced some type of health complaint during the past 30 days, and 78% of them sought treatment. Using the cut-off as described above, the prevalence of CMD was 14%. Even considering the reduction of the SRQ-20 to 19 items, the prevalence of CMD is much higher than that found in the Ministry of Health's 2018 report.^21^

**Table 1:** Summary statistics for the sample population

	Mean/Frequency	s.d.	Respondents, *n*
Female	61.74%	0.4862	1490
Age, years	39.93	16.5041	1490
Highest grade completed (in year)	9.99	3.0966	1489
In work/employment	40.00%	0.4901	1480
Any health complaints^a^	43.30%	0.4957	1485
Sought treatment	78.28%	0.4127	640
SRQ-20 score	2.56	2.9601	1321
SRQ-20 ≥ 6	13.63%	0.3432	1321

SQR-20, 20-item Self Reporting Questionnaire.

a. All self-reported incidence of any health complaints over the past 30 days. Only participants with health complaints were asked whether they sought treatment.

**Fig. 2:**
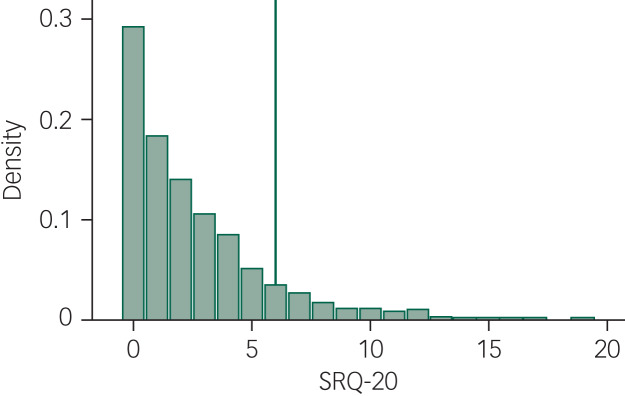
Distribution of scores on the 20-item Self Reporting Questionnaire (SRQ-20). The vertical line marks the cut-off for common mental disorders (CMD) used in this study (SRQ-20 ≥ 6).

Figure 2 depicts the distribution of the SRQ-20 scores: they are quite smoothly distributed and have a high internal consistency (Cronbach's alpha: 0.813).

We found significant differences in the measure of mental distress across gender, age and education (Table 2). Female, older and less educated individuals experienced higher scores than did other population groups. These results were similar to previous findings in Indonesia.^12–14^ As these three characteristics were presumably unaffected by mental distress in the past 30 days, we used them as controls in our subsequent analyses.

**Table 2:** Scores on the 20-item Self Reporting Questionnaire (SRQ-20) by gender, age and education

	Mean	s.d.	*n*	*P*^a^
Total	2.559	2.960	1321	
Gender				<0.001
Male	1.757	2.429	511	
Female	3.064	3.149	810	
Age, years				<0.001
17–25	1.954	2.467	304	
26–45	2.163	2.690	534	
46–65	3.016	3.149	380	
65+	4.709	3.648	103	
Education				<0.001
None	4.310	3.873	42	
Primary	3.471	3.474	189	
Lower secondary	2.675	2.964	249	
Upper secondary	2.370	2.708	560	
Tertiary	1.961	2.672	280	

a. *P*-values were obtained from an *F*-test of joint significance when regressing the instrument on the respective categories, clustering standard errors at the household level.

### Sociodemographic groups affected by CMD

**Fig. 3:**
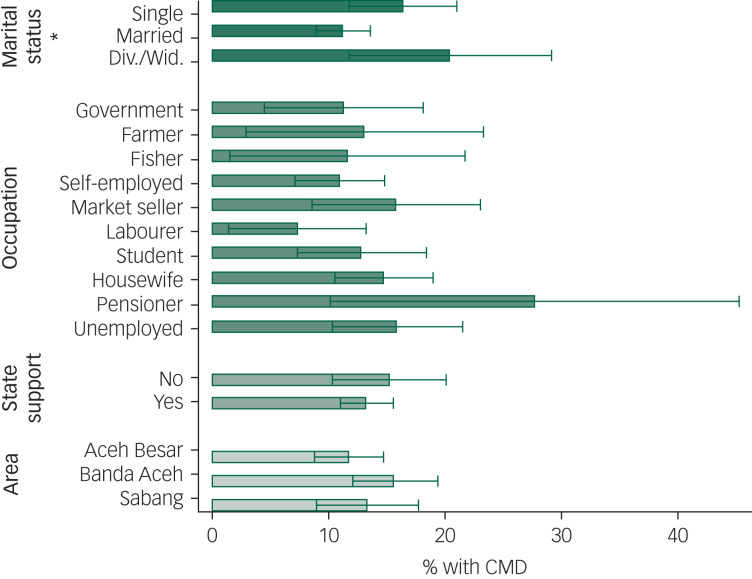
Predicted probabilities of common mental disorders (CMD) by marital status, occupation, state support and study area. Predicted probabilities were obtained by employing a linear probability model regressing the indicator of CMD (i.e. SRQ-20 ≥ 6) on the characteristic of interest controlling for gender, age and education. The model was then used to predict probabilities over each category of the characteristic of interest. Standard errors were clustered at the household level; 95% confidence intervals are displayed. Asterisks indicate significant differences between categories, with *P < 0.05. Div., divorced; Wid., widowed. Numerical results are shown in supplementary Table 3.

We predicted the probability for CMD (SRQ-20 ≥ 6) using a linear probability model controlling for age, gender and educational level. Figure 3 compares the predicted probabilities by marital status, occupation, study area and presence in households receiving state support. We found a significant difference in predicted probabilities of CMD by marital status. On average, widowed and divorced participants had a 1.8-times higher predicted probability for CMD compared with married participants. Although there were significant differences between single occupation groups, these differences were not jointly significant over all occupation groups; there were also no significant differences by reception of state support or study area.

### Family functioning and CMD

We took a closer look at the role of families and CMD. Table 3 displays summary statistics by household. In 26% of all households, at least one of the interviewed members showed symptoms of CMD; moreover, 15% of all interviewed household heads had symptoms of CMD.

**Table 3:** Family characteristics of the study sample by household

	Mean/Frequency	s.d.	Observations, *n*
Number of household members	4.56	1.92	632
Number of conducted interviews	2.36	1.47	632
Any members with CMD	25.64%	0.44	589
Number of members with CMD	0.31	0.58	589
Head was interviewed	65.51%	0.48	632
Head with CMD	14.68%	0.35	361

CMD, common mental disorders.

**Fig. 4:**
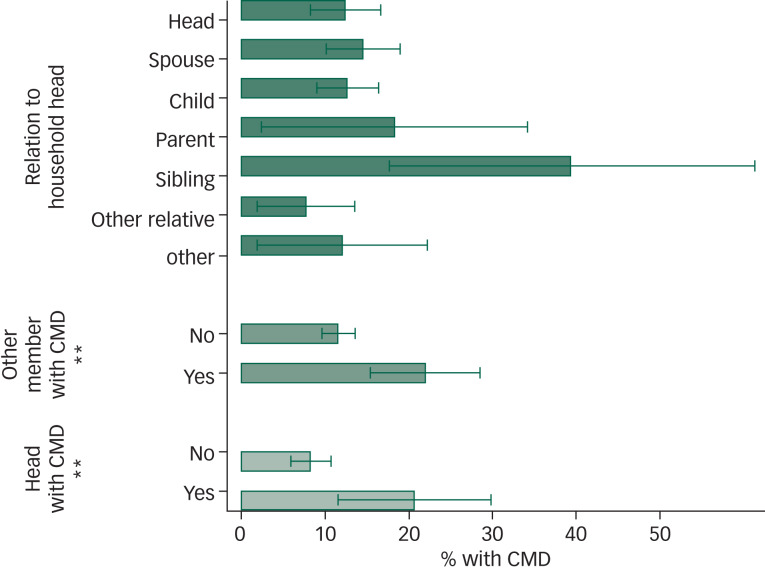
Predicted probabilities of common mental disorders (CMD) by family characteristics. Predicted probabilities were obtained by employing a linear probability model regressing the indicator of CMD (i.e. SRQ-20 ≥ 6) on the characteristic of interest controlling for gender, age and education. The model was then used to predict probabilities over each category of the characteristic of interest. Standard errors were clustered at the household level; 95% confidence intervals are displayed. Asterisks indicate significant differences between categories, with **P < 0.01. Numerical results are shown in supplementary Table 4.

As displayed in Fig. 4, there was a significantly higher share of CMD among siblings of household heads as compared with the spouses, children, other relatives of the head or the heads themselves. Overall, the differences were not jointly significant. Respondents were about twice as likely to show symptoms of CMD if any other household member had CMD or if the household head had CMD.

**Fig. 5:**
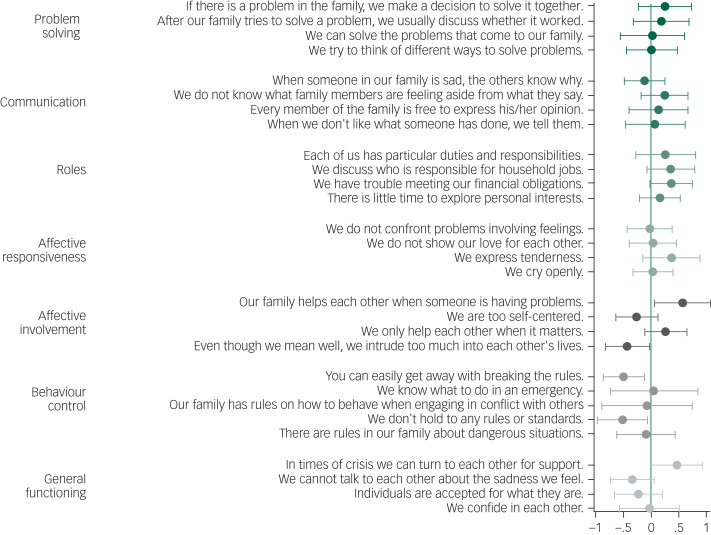
Correlation of common mental disorders (CMD) with responses to McMaster Family Assessment Device (FAD) items. Coefficient estimates from an ordered logistic model regressing FAD items on the CMD indicator (i.e. SRQ-20 ≥ 6) controlling for gender, age and education. Standard errors are clustered at the household level. The answer scale ranges from 1, strongly disagree to 4, strongly agree. Positive coefficients indicate a higher likelihood to agree with a statement; 95% confidence intervals are displayed. Numerical results and marginal effects are shown in supplementary Table 5.

We used an ordered logistic regression model to analyse the association of mental distress with family functioning as measured by the FAD. Figure 5 displays the coefficient estimates, with positive coefficients indicating a higher propensity to agree with the specific statement. Marginal effects are reported in supplementary Table 5.

People with CMD were significantly more likely to perceive their families as performing well regarding several aspects in the dimensions of affective involvement and behaviour control. This is somewhat surprising, considering findings from the wider literature, which indicate that mental disorders are negatively correlated with family functioning.^6^

### Costs associated with CMD

We next turned to the possible financial burden of mental distress by investigating its association with working status, working hours, working days, monthly pay and disturbance of daily tasks. To estimate the association of mental distress, we regressed the outcomes of interest on the binary indicator for CMD and predicted the outcome of interest by CMD status. Associations with work characteristics are shown in Table 4. Working status, working days, working hours and earnings were not significantly associated with CMD.

**Table 4:** Differences in work characteristics^a^

	SRQ-20 ≥ 6			
	No	Yes	*P*	Observations, *n*
Is working	0.42 (0.01)	0.35 (0.03)	0.061	1313
Working days/week	5.87 (0.05)	5.69 (0.21)	0.422	519
Working hours/week	32.13 (1.09)	35.04 (3.50)	0.419	504
Log of earnings	14.29 (0.04)	13.99 (0.16)	0.068	520
Daily tasks affected	0.07 (0.01)	0.29 (0.04)	<0.001**	1308
Affected days	0.28 (0.05)	1.78 (0.32)	<0.001**	1311
Sought treatment owing to feelings	0.07 (0.01)	0.26 (0.04)	<0.001**	1316
Feelings caused by physical problems	0.02 (0.00)	0.24 (0.03)	<0.001**	1314

SQR-20, 20-item Self Reporting Questionnaire.

a. Predicted outcomes for each characteristic of interest are obtained by regressing the characteristic of interest on the common mental disorders (CMD) indicator (i.e. SRQ-20 ≥ 6) controlling for gender, age and education. Then, the model is used to predict outcomes by CMD status. Standard errors (shown in parentheses) are clustered at the household level. Asterisks indicate significant differences between categories, with **P < 0.01.

Mental distress in the form of negative feelings can substantially affect work productivity. Participants with elevated mental distress were four times more likely to report any impact of negative feelings on their work than did their counterparts (*P* < 0.01). Over the past 30 days, an average of 1.5 more days were affected than was the case for their counterparts (*P* < 0.01). They were nearly four times more likely to seek treatment owing to these feelings (*P* < 0.01); however, they also more often reported that physical problems were the main cause of these feelings (*P* < 0.01).

Finally, we examined the differences in health complaints and treatments sought for individuals with CMD; the results are depicted in Table 5. Individuals with mental distress were twice as likely to have had a health complaint during the past 30 days (*P* < 0.01) and 1.8 times (*P* < 0.05) more likely to attend private practices than did their counterparts. There were no economically meaningful differences in time spent seeking treatment, but people with CMD (*P* < 0.05) paid on average twice the treatment costs of their counterparts.

**Table 5:** Differences in healthcare seeking^a^

	SRQ-20 ≥ 6			
	No	Yes	*P*	Observations, *n*
Any health complaint	0.349 (0.015)	0.681 (0.031)	<0.001**	1317
Sought treatment	0.757 (0.023)	0.823 (0.033)	0.095	517
Number of facilities	1.119 (0.022)	1.190 (0.043)	0.147	398
Primary health centre	0.455 (0.034)	0.487 (0.047)	0.580	398
Government clinic	0.142 (0.023)	0.162 (0.037)	0.646	398
Private clinic	0.027 (0.012)	0.020 (0.012)	0.634	398
Private practice	0.141 (0.024)	0.248 (0.041)	0.020*	398
Joint clinic/practice	0.247 (0.028)	0.211 (0.040)	0.441	398
Traditional healer	0.049 (0.014)	0.020 (0.016)	0.212	398
Travel time, min	15.926 (0.907)	16.623 (1.793)	0.720	376
Waiting time, min	29.370 (4.478)	29.654 (5.782)	0.970	374
Treatment time, min	10.696 (0.755)	13.471 (1.473)	0.101	375
Travel costs, k IDR	17.125 (2.827)	28.936 (7.601)	0.157	377
Treatment costs, k IDR	23.702 (4.640)	49.500 (11.435)	0.024*	373
Medication costs, k IDR	52.390 (14.341)	63.792 (17.172)	0.623	377

SQR-20, 20-item Self Reporting Questionnaire; k IDR, thousand Indonesian rupiah.

a. Predicted outcomes for each characteristic of interest were obtained by regressing the characteristic of interest on the common mental disorders (CMD) indicator (i.e. SRQ-20 ≥ 6) controlling for gender, age and education. Then, the model is used to predict outcomes by CMD status. Standard errors (shown in parentheses) are clustered at the household level. Asterisks indicate significant differences between categories, with *P < 0.05, **P < 0.01.

## Discussion

Common mental disorders constitute a substantial share of the global burden of disease. Previous evidence from LMICs suggests that disadvantaged socioeconomic groups might be especially vulnerable to CMD. Moreover, studies from high-income countries show that people with CMD can face considerably higher direct and indirect costs than their non-affected counterparts. Although there is evidence of a correlation between CMD and poverty proxies in LMICs, analyses of associated costs are still rare.^10^

In our study, we used a population-based household survey to identify the affected groups and the associated financial and healthcare costs in Aceh, Indonesia. Using the SRQ-20, we found a prevalence of common mental disorders of 14% in our study sample, with higher scores among female, older and less educated participants. Widowed and divorced respondents were twice as likely as married respondents to have CMD. The association of CMD with gender, education and marital status confirms findings from other low- and middle-income settings and in Indonesia.^11–14,32^ However, evidence on the gradient of age is very mixed in other studies from Indonesia: our results match the findings from Das et al (2007)^33^ but contradict findings by Peltzer & Pengpid (2018)^15^ that showed higher CMD in younger respondents. In India, respondents below 30 years of age were at significantly lower risk for depressive disorders, but differences between older age cohorts were not significant.^32^ The World Mental Health Surveys did not find any age gradient for LMICs.^34^

We also found that the probability of CMD nearly doubled when another household member showed symptoms of CMD, which is in line with the literature.^33^ Surprisingly, our results show that individuals with CMD reported better family functioning regarding affective involvement and behaviour control compared with their unaffected counterparts. This contradicts findings from studies in other countries;^6–8,35^ it is unclear whether this difference stems from the fact that our study is population-based, whereas all other studies compare clinical and non-clinical samples or only consider the affected population. Further studies on a similar topic might consider comparing the condition of the participants in clinical and community settings.

In line with the literature, our study indicates that people with CMD are affected in their productivity and physical well-being.^4,5,9–12,35,36^ Their daily activities are more severely disturbed by negative emotions, but this is attributed to physical problems; in addition, they are twice as likely to have health complaints. Compared with other people who sought treatment, participants with CMD faced twice the treatment costs. Independent of the direction of causality, this signals a vulnerability of people with mental distress to poverty; indeed, a correlation between poverty and CMD is very common in LMICs.^10^ Although disentangling the causal directions is challenging, longitudinal evidence suggests that both might interact in a vicious cycle.^37^ This would exacerbate any excess costs found in cost-of-illness studies. These costs also transform to the societal level: the societal costs of mental distress were estimated in 2010 to be 1.5% of the annual global gross domestic product (GDP).^38^ Nevertheless, treatment possibilities can be comparatively cheap: cost-effective interventions for mental, neurological and substance use disorders are estimated to cost US$3–4 per capita per year in LMICs.^39^ Moreover, previous studies showed that mental health interventions can effectively break the vicious cycle of poverty and mental illness.^40^ Policies to combat the burden of mental disorders are thus economically feasible, and policy stakeholders should react to decrease the individual and societal economic burden of mental distress.

### Limitations

A few limitations are noteworthy: our analyses focus on associations instead of causalities. For example, it is unclear whether CMD cause lower productivity, or whether lower productivity gives rise to CMD, or both. Moreover, elevated mental distress also coincides with the occurrence of general health problems, and negative emotions are reported to be caused by physical problems. This raises the question of whether the SRQ-20 proxies physical health rather than mental health. This is difficult to disentangle, as several of the items ask for symptoms that could also have physical causes. On the other hand, the association of physical with mental conditions has been shown frequently in clinical settings and population-wide;^5^ again, the direction of causality is not always clear.

Our data are additionally constrained in several dimensions. First, despite the random sampling, female and unemployed participants are overrepresented in our sample. We partly adjusted for this by controlling for gender, age and education, but household members who did not participate (mostly owing to absence) might show a systematically different burden of mental distress. Still, as on average more than 70% of all adult household members participated in the survey, we are confident that the loss of representativeness in the targeted study sample is not too large. Second, our study took place in an urban and peri-urban setting; treatment costs might be higher and labour market options less diverse in more remote areas, potentially changing the cost pattern of mental distress.

Finally, the province of Aceh might not be representative of all Indonesian regencies. It is one of five provinces with special autonomy rights and the only regency in Indonesia where Sharia law is in place. The region was the setting of a long conflict between autonomous groups and the central government in Jakarta, and it was devastated by the Indian Ocean tsunami in 2004. Although Indonesia is frequently hit by natural disasters, this tsunami stood out in terms not only of casualties and destruction, but also inflow of international aid. This context might have caused a higher prevalence of mental distress compared with other regions but might also have created more opportunities to cope with the shock compared with other disasters.

### Implications

Our finding that people from disadvantaged groups are significantly more likely to have common mental disorders stresses the importance of low-threshold and affordable mental healthcare. Despite different policy efforts in Aceh in the past years, the prevalence of mental distress is substantial. In light of a double burden of physical and psychological conditions, accessible, adequate and financially feasible healthcare is important, independent of the direction of causality. Although Indonesia is on its way to establishing universal healthcare, the difference in treatment costs for people with mental distress is still considerable; more efforts to decrease out-of-pocket payments are needed. Also, to the extent that mental illness affects work productivity, early detection and treatment are important to prevent financial strains during the progression of the disorder. Although the causality might run both ways, this vicious cycle can be broken through mental health interventions. Putting mental health on the policy agenda can thus yield improvements in the dimensions of mental, physical and financial well-being.

## Data Availability

Owing to the restrictions related to protection of participants’ privacy, the data that support the findings of this study are not publicly available. The data are available only from the corresponding author (M.M.), on reasonable request.

## Supplementary material

For supplementary material accompanying this paper visit http://dx.doi.org/10.1192/bjo.2020.108. click here to view supplementary material
