# Supplementary material for: Mental distress and its association with sociodemographic and economic characteristics: community-based household survey in Aceh, Indonesia
Source: BJPsych Open. 2020 Nov 4;6(6):e134. doi: 10.1192/bjo.2020.108 (PMC7745230; doi:10.1192/bjo.2020.108)
Supplement: Supplementary file 1 [file bjosup.zip › S2056472420001088sup002.docx]

# Appendix

**Table 1. Summary statistics for each screening item**

|  | **Mean** | **SD** | **Min** | **Max** | **p50** | **N** |
| --- | --- | --- | --- | --- | --- | --- |
| **SRQ-20** |  |  |  |  |  |  |
| Headaches | 0.4015 | 0.4904 | 0 | 1 | 0 | 1487 |
| Poor appetite | 0.2349 | 0.4241 | 0 | 1 | 0 | 1477 |
| Sleeping badly | 0.2618 | 0.4398 | 0 | 1 | 0 | 1478 |
| Easily frightened | 0.1039 | 0.3053 | 0 | 1 | 0 | 1472 |
| Hands shaking | 0.0819 | 0.2743 | 0 | 1 | 0 | 1478 |
| Nervous, tense, worried | 0.1671 | 0.3732 | 0 | 1 | 0 | 1454 |
| Poor digestion | 0.2186 | 0.4135 | 0 | 1 | 0 | 1482 |
| Trouble thinking clearly | 0.0665 | 0.2493 | 0 | 1 | 0 | 1458 |
| Feeling unhappy | 0.0605 | 0.2385 | 0 | 1 | 0 | 1455 |
| Crying more often | 0.0483 | 0.2145 | 0 | 1 | 0 | 1469 |
| Difficult enjoying activities | 0.0807 | 0.2724 | 0 | 1 | 0 | 1475 |
| Difficult making decisions | 0.0516 | 0.2213 | 0 | 1 | 0 | 1473 |
| Daily work suffering | 0.1141 | 0.3181 | 0 | 1 | 0 | 1472 |
| Unable to play useful part | 0.0266 | 0.1611 | 0 | 1 | 0 | 1464 |
| Lost interest in things | 0.0534 | 0.2249 | 0 | 1 | 0 | 1424 |
| Feeling worthless | 0.0179 | 0.1326 | 0 | 1 | 0 | 1454 |
| Feeling always tired | 0.2066 | 0.4050 | 0 | 1 | 0 | 1476 |
| Easily tired | 0.2268 | 0.4189 | 0 | 1 | 0 | 1468 |
| Uncomfortable feelings in stomach | 0.2686 | 0.4434 | 0 | 1 | 0 | 1478 |
|  | | | | | | |

**Table 2. Outcome variables**

|  | **Mean** | **S.D.** | **N** |
| --- | --- | --- | --- |
| **Categorical variables** |  |  |  |
| Marital status |  |  |  |
| *Never married* | 0.27 |  | 397 |
| *Married* | 0.62 |  | 920 |
| *Divorced/Widowed* | 0.12 |  | 172 |
| Occupation code |  |  |  |
| *Government* | 0.05 |  | 79 |
| *Farmer* | 0.05 |  | 75 |
| *Fisher* | 0.02 |  | 32 |
| *Self-employed* | 0.16 |  | 244 |
| *Market seller* | 0.08 |  | 126 |
| *Laborer* | 0.04 |  | 57 |
| *Student* | 0.07 |  | 108 |
| *Housewife* | 0.34 |  | 508 |
| *Pensioner* | 0.02 |  | 35 |
| *Unemployed* | 0.13 |  | 192 |
| Area |  |  |  |
| *Aceh Besar* | 0.42 |  | 622 |
| *Banda Aceh* | 0.38 |  | 568 |
| *Sabang* | 0.20 |  | 300 |
| Relation to head |  |  |  |
| *Head* | 0.28 |  | 420 |
| *Spouse* | 0.30 |  | 445 |
| *Child* | 0.30 |  | 449 |
| *Parent* | 0.04 |  | 65 |
| *Sibling* | 0.02 |  | 29 |
| *Other relative* | 0.04 |  | 55 |
| *Other* | 0.02 |  | 24 |
| **Continuous variables** |  |  |  |
| Any state support | 0.79 | 0.41 | 1,490 |
| Any member with CMD* | 0.26 | 0.44 | 589 |
| Head with CMD* | 015 | 0.35 | 361 |
| Is working | 0.40 | 0.49 | 1,480 |
| Working days per week | 5.88 | 1.14 | 575 |
| Working hours per week | 32.26 | 20.28 | 557 |
| Log of earnings | 14.25 | 0.86 | 572 |
| Daily tasks affected | 0.10 | 0.30 | 1,468 |
| Affected days | 0.50 | 2.33 | 1,472 |
| Sought treatment due to feelings | 0.09 | 0.29 | 1,478 |
| Feelings caused by physical problems | 0.06 | 0.24 | 1,469 |
| Any health complaints | 0.43 | 0.50 | 1,485 |
| Sought treatment | 0.78 | 0.41 | 640 |
| Number of facilities visited | 1.13 | 0.34 | 497 |
| Community health center | 0.43 | 0.50 | 497 |
| Government clinic | 0.16 | 0.37 | 497 |
| Private clinic | 0.02 | 0.15 | 497 |
| Private practice | 0.17 | 0.37 | 497 |
| Joint clinic/practice | 0.24 | 0.43 | 497 |
| Traditional healer | 0.05 | 0.21 | 497 |
| Travel time [min] | 17.70 | 35.87 | 468 |
| Waiting time [min] | 29.06 | 61.69 | 466 |
| Treatment time [min] | 11.60 | 13.31 | 466 |
| Travel costs [k IDR] | 21.16 | 68.36 | 468 |
| Treatment costs [k IDR] | 27.78 | 79.86 | 464 |
| Medication costs [k IDR] | 50.74 | 183.43 | 469 |
| * Collapsed by household. |  |  |  |

**Table 3. Predicted probabilities by marital status, occupation, state support and area**

|  | **Predicted probability** |  | **Predicted probability** |
| --- | --- | --- | --- |
| **Marital status** |  | **Occupation** |  |
| Never married | 0.164 | Government | 0.113 |
|  | [0.117,0.210] |  | [0.046,0.181] |
| Married | 0.113 | Farmer | 0.131 |
|  | [0.089,0.136] |  | [0.029,0.233] |
| Div./Wid. | 0.204 | Fisher | 0.116 |
|  | [0.117,0.291] |  | [0.015,0.217] |
| **State support** |  | Self-employed | 0.110 |
| No | 0.151 |  | [0.071,0.148] |
|  | [0.102,0.201] | Market seller | 0.158 |
| Yes | 0.132 |  | [0.086,0.230] |
|  | [0.110,0.155] | Laborer | 0.073 |
| **Area** |  |  | [0.014,0.132] |
| Aceh Besar | 0.118 | Student | 0.129 |
|  | [0.088,0.147] |  | [0.073,0.184] |
| Banda Aceh | 0.157 | Housewife | 0.147 |
|  | [0.120,0.193] |  | [0.105,0.190] |
| Sabang | 0.134 | Pensioner | 0.277 |
|  | [0.090,0.177] |  | [0.101,0.452] |
|  |  | Unemployed | 0.159 |
|  |  |  | [0.104,0.214] |
| Predicted probabilities are obtained by employing a linear probability model regressing the indicator of CMD (i.e. SRQ-20>=6) on the characteristic of interest controlling for sex, age and education. Then, the model is used to predict probabilities over each category of the characteristic of interest. Standard errors are clustered at the household level. 95% confidence intervals in square brackets. | | | |

**Table 4. Predicted probabilities by family characteristics**

|  | **Predicted probability** |  | **Predicted probability** |
| --- | --- | --- | --- |
| **Relation to household head** |  | **Any other member with CMD** |  |
| Head | 0.125 | No | 0.116 |
|  | [0.083,0.167] |  | [0.096,0.136] |
| Spouse | 0.146 | Yes | 0.220 |
|  | [0.102,0.189] |  | [0.155,0.285] |
| Child | 0.127 | **Head with CMD** |  |
|  | [0.090,0.164] | No | 0.084 |
| Parent | 0.183 |  | [0.060,0.107] |
|  | [0.025,0.342] | Yes | 0.207 |
| Sibling | 0.393 |  | [0.115,0.298] |
|  | [0.176,0.611] |  |  |
| Other relative | 0.078 |  |  |
|  | [0.019,0.136] |  |  |
| Other | 0.121 |  |  |
|  | [0.020,0.222] |  |  |
| Predicted probabilities are obtained by employing a linear probability model regressing the indicator of CMD on the characteristic of interest controlling for sex, age and education. Then, the model is used to predict probabilities over each category of the characteristic of interest. Standard errors are clustered at the household level. 95% confidence intervals in square brackets. | | | |

**Table 5. Marginal effects of CMD on FAD items**

|  | **Total** | **Strongly disagree** | **Disagree** | **Agree** | **Strongly agree** |
| --- | --- | --- | --- | --- | --- |
| If there is a problem in the family, we make a decision to solve it together | 0.2598 | -0.0015 | -0.0022 | -0.0323 | 0.0360 |
|  | (0.2456) | (0.0014) | (0.0020) | (0.0328) | (0.0359) |
| After our family tries to solve a problem, we usually discuss whether it worked. | 0.1864 | -0.0004 | -0.0018 | -0.0186 | 0.0208 |
|  | (0.2571) | (0.0006) | (0.0024) | (0.0269) | (0.0297) |
| We can solve the problems that come to our family. | 0.0310 |  | -0.0007 | -0.0022 | 0.0029 |
|  | (0.2983) |  | (0.0063) | (0.0218) | (0.0281) |
| We try to think of different ways to solve problems. | 0.0155 | -0.0000 | -0.0012 | 0.0001 | 0.0011 |
|  | (0.2360) | (0.0002) | (0.0182) | (0.0011) | (0.0173) |
| When someone in our family is sad, the others know why. | -0.1107 | 0.0015 | 0.0188 | -0.0138 | -0.0065 |
|  | (0.1853) | (0.0027) | (0.0320) | (0.0241) | (0.0107) |
| We do not know what family members are feeling aside from what they say. | 0.2494 | -0.0016 | -0.0353 | 0.0260 | 0.0110 |
|  | (0.2149) | (0.0014) | (0.0288) | (0.0197) | (0.0106) |
| Every member of the family is free to express his/her opinion. | 0.1373 |  | -0.0009 | -0.0177 | 0.0186 |
|  | (0.2686) |  | (0.0017) | (0.0359) | (0.0376) |
| When we don't like what someone has done, we tell them. | 0.0687 |  | -0.0024 | -0.0049 | 0.0073 |
|  | (0.2757) |  | (0.0092) | (0.0207) | (0.0299) |
| Each of us has particular duties and responsibilities | 0.2607 | -0.0004 | -0.0112 | -0.0108 | 0.0224 |
|  | (0.2740) | (0.0005) | (0.0111) | (0.0141) | (0.0253) |
| We discuss who are responsible for household jobs. | 0.3611 | -0.0018 | -0.0440 | 0.0220* | 0.0238 |
|  | (0.2191) | (0.0012) | (0.0243) | (0.0106) | (0.0163) |
| We have trouble meeting our financial obligations. | 0.3672 | -0.0234* | -0.0616 | 0.0597 | 0.0252 |
|  | (0.1950) | (0.0114) | (0.0344) | (0.0310) | (0.0148) |
| There is little time to explore personal interests. | 0.1643 | -0.0057 | -0.0350 | 0.0387 | 0.0019 |
|  | (0.1892) | (0.0062) | (0.0407) | (0.0445) | (0.0024) |
| We do not confront problems involving feelings. | -0.0216 | 0.0002 | 0.0038 | -0.0034 | -0.0005 |
|  | (0.2058) | (0.0017) | (0.0365) | (0.0330) | (0.0052) |
| We do not show our love for each other. | 0.0349 | -0.0023 | -0.0044 | 0.0062 | 0.0005 |
|  | (0.2171) | (0.0141) | (0.0278) | (0.0388) | (0.0030) |
| We express tenderness. | 0.3736 | -0.0003 | -0.0141 | -0.0208 | 0.0351 |
|  | (0.2632) | (0.0003) | (0.0087) | (0.0193) | (0.0278) |
| We cry openly. | 0.0403 | -0.0016 | -0.0081 | 0.0087 | 0.0011 |
|  | (0.1850) | (0.0073) | (0.0373) | (0.0396) | (0.0049) |
| Our family helps each other when someone is having problems. | 0.5753* | -0.0011 | -0.0066* | -0.0593 | 0.0670 |
|  | (0.2594) | (0.0007) | (0.0029) | (0.0322) | (0.0349) |
| We are too self-centered. | -0.2510 | 0.0468 | -0.0295 | -0.0157 | -0.0016 |
|  | (0.1949) | (0.0380) | (0.0257) | (0.0114) | (0.0013) |
| We only help each other when it matters. | 0.2699 | -0.0250 | -0.0379 | 0.0550 | 0.0079 |
|  | (0.1923) | (0.0166) | (0.0295) | (0.0394) | (0.0065) |
| Even though we mean well, we intrude too much into each other’s lives. | -0.4234* | 0.0791 | -0.0349 | -0.0422* | -0.0021 |
|  | (0.2051) | (0.0415) | (0.0233) | (0.0183) | (0.0012) |
| You can easily get away with breaking the rules. | -0.4929** | 0.0888* | -0.0583* | -0.0277** | -0.0027* |
|  | (0.1902) | (0.0375) | (0.0280) | (0.0096) | (0.0012) |
| We know what to do in an emergency. | 0.0540 | -0.0003 | -0.0028 | 0.0018 | 0.0013 |
|  | (0.4045) | (0.0025) | (0.0204) | (0.0130) | (0.0100) |
| Our family has rules on how to behave when engaging in conflict with others | -0.0721 | 0.0002 | 0.0029 | -0.0012 | -0.0020 |
|  | (0.4173) | (0.0014) | (0.0173) | (0.0076) | (0.0112) |
| We don’t hold to any rules or standards. | -0.5157* | 0.0645 | 0.0186* | -0.0803* | -0.0028* |
|  | (0.2328) | (0.0343) | (0.0077) | (0.0321) | (0.0014) |
| There are rules in our family about dangerous situations. | -0.0904 | 0.0002 | 0.0073 | -0.0033 | -0.0042 |
|  | (0.2703) | (0.0005) | (0.0223) | (0.0107) | (0.0121) |
| In times of crisis we can turn to each other for support. | 0.4628 | -0.0003 | -0.0046* | -0.0488 | 0.0537 |
|  | (0.2397) | (0.0003) | (0.0023) | (0.0293) | (0.0313) |
| We cannot talk to each other about the sadness we feel. | -0.3325 | 0.0111 | 0.0654 | -0.0744 | -0.0021 |
|  | (0.2018) | (0.0079) | (0.0373) | (0.0438) | (0.0013) |
| Individuals are accepted for what they are. | -0.2255 | 0.0002 | 0.0014 | 0.0382 | -0.0397 |
|  | (0.2239) | (0.0003) | (0.0016) | (0.0359) | (0.0376) |
| We confide in each other. | -0.0299 |  | 0.0005 | 0.0036 | -0.0041 |
|  | (0.2789) |  | (0.0045) | (0.0335) | (0.0380) |
| Coefficient estimates and marginal effects from an ordered logistic model regressing FAD items on the CMD indicator controlling for sex, age and education. Standard errors are clustered at the household level. The answer scale ranges from 1 – Strongly disagree to 4 – Strongly agree. Positive coefficients indicate a higher likelihood to agree to a statement, and vice versa. Stars indicate significant differences between categories, with * p<0.05, ** p<0.01. | | | | | |

______________________________________

i Due to feasibility, the following districts of Aceh Besar were excluded: Pulo Aceh, Lhoong, Lembah Seulawah, Leupung and Kota Jantho.

ii Members are those identified by the key informant of the household as members.
